# Aneurysm

**Published:** 1908-04-25

**Authors:** 


### Points in Surgery.

### ANEURYSM.

### CONSTITUTIONAL TREATMENT.

The ideal treatment of an aneurysm is to ligature the vessel above and below and remove the sac. Such a radical proceeding is, however, only possible in rare cases, where the aneurysm is in an accessible site. In the majority, generally for anatomical reason, some more palliative method of treatment must be adopted. If, for instance, the arch of the aorta or any of the large intra-thoracic trunks are affected, it is obvious that surgical interference is impracticable. Here reliance must be placed upon constitutional treatment, the object of which is to render the condition of the patient as favourable as possible for the occurrence of a natural cure. Thus the patient must be kept absolutely at rest: this encourages intravascular clotting by slowing the circulation and diminishing the force of the heart's action. This object is also attained by restricting the patient's diet, both fluid and solid, particularly the former. Tufnel, the physician who first pointed out the importance of this, and from whom the method derives its name, went so far as to say that not more than half a pint of fluid should be allowed in the twenty-four hours. This is an extreme measure which patients often find exceedingly irksome. The rationale of the treatment is quite sound, since anything in the nature of plethora must obviously be inimical to spontaneous cure. Each case should be studied individually, and the treatment pushed only as far as the patient can comfortably stand. Extreme measures will probably do more harm than good by causing him to fret. In combination with restriction of the diet, venesection was often practised in the old days, a method associated with the name of Valsalva—the idea being to deplete still further the tension in the vascular system. It is not often done nowadays. Venesection has suffered from a reaction. Our predecessors regarded it as a panacea for most mortal ills, and had recourse to it indiscriminately. In fact, in the early half of last century it was no uncommon thing for a man to be bled periodically whether he was ailing or not; and farmers bringing in their cattle to market at the county town would seize the opportunity of visiting the apothecary to have some blood let. It is not unnatural that any system, thus applied without discrimination, should fall into disuse; and instead of being regarded as a panacea venesection came to be looked upon as a useless, if not dangerous, procedure. The truth lies somewhere between the two extremes, for there can be no doubt that for some of the conditions in the province of the physician venesection is a measure of value. But the fact remains that as a means of treating aneurysm it has fallen almost entirely into abeyance.

Free purgation has been advocated as being of assistance in the same way. That it reduces a tendency to plethora there can be no doubt; and it has this advantage over the other forms of treatment already mentioned, that even if it produces no benefit, it is not at all likely to harm the patient.

Of drugs most reliance is placed upon iodide of potassium, not necessarily because the aneurysm is of syphilitic origin (and, as has been pointed out, the great majority of aneurysms are due directly or indirectly to syphilis), but because it certainly has a depressing effect on the heart. This is so well recognised that in giving potassium iodide for syphilis it is, or should be, combined with some form of stimulant. Sal volatile is, perhaps, as good as any. It has been suggested that the depressing effect of the drug is due to the potassium, and the use of sodium iodide has been advised instead of, or combined with, the potassium salt; but the comparative rarity with which sodium iodide is prescribed goes to show that the statement does not rest on a very solid basis.

A patient who is being kept completely at rest in bed and treated with potassium iodide at the same time is not unlikely to present symptoms of iodism, for the reason that his excretory apparatus is working at very low pressure. The prominent signs of this are the appearance of coryza and of an acneform eruption. The former is explained by the fact that some, at any rate, of the iodide given is excreted by the respiratory mucous membrane (hence the value of the drug in a dry chronic bronchitis). One often hears it said that if symptoms of iodism occur they will disappear if the dose is doubled. This is a fallacy which is as common as it is easy to explain. Iodide is a salt which must be given with plenty of water if it is to be absorbed; and it will be found that if the patient takes half a pint of water with each dose of the drug iodism can nearly always be avoided.

Another drug which has come to the front lately in the treatment of aneurysm is calcium chloride. That it has the power of increasing the coagulability of the blood is demonstrated by the success of its administration in purpuric conditions and in articaria, as well as in that homely but irritating malady chilblains. It has been shown that chilblains are as often due to diminished as to increased coagulability of the blood; and when they are due to this cause a little calcium chloride will often cause them to disappear like magic.

These are the main lines of constitutional treatment for aneurysm. But it will be seen that they are not a certain means of cure, but rather attempts to bring about a set of conditions which may favour spontaneous cure by intra-vascular clotting. The best that can be said of them is that they tend to prolong the patient's life; but in prolonging it there can be no doubt they also make it a misery to him. So that it is really an open question whether such measures are of any value. If a patient is found to be suffering from inoperable aneurysm the situation should be explained to him, and it should be left to him to decide whether he would prefer "a short life and a merry one," or whether he would submit to irksome and tedious treatment on the chance of prolonging it.

